# mRNA isoform switches during mouse zygotic genome activation

**DOI:** 10.1111/cpr.13655

**Published:** 2024-05-19

**Authors:** Fan Li, Najmeh Karimi, Siqi Wang, Tianshi Pan, Jingxi Dong, Xin Wang, Sinan Ma, Qingtong Shan, Chao Liu, Ying Zhang, Wei Li, Guihai Feng

**Affiliations:** ^1^ State Key Laboratory of Stem Cell and Reproductive Biology Institute of Zoology, Chinese Academy of Sciences Beijing China; ^2^ Key Laboratory of Organ Regeneration and Reconstruction Chinese Academy of Sciences Beijing China; ^3^ University of Chinese Academy of Sciences Beijing China; ^4^ Beijing Institute for Stem Cell and Regenerative Medicine Beijing China; ^5^ College of Life Sciences Northeast Agricultural University Harbin China; ^6^ Medical School University of Chinese Academy of Sciences Beijing China

Dear Editor,

Zygotic genome activation (ZGA) is the first transcriptional event following fertilization in mammals. ZGA is a coordinated process of the degradation of maternal mRNA stored in the oocyte and the transcription of new mRNAs from the zygotic genome.^1^, ^2^, ^3^ In mice, ZGA can be divided into two stages: minor ZGA, occurring at the one‐cell stage and activating a limited number of genes, and major ZGA, occurring during the mid to late 2‐cell stage and initiating the comprehensive transcriptional activation of the zygotic genome.^1^, ^3^, ^4^ Alternative splicing is a conserved and important regulatory mechanism to increase the diversity of mRNA isoforms, which allows a single gene to generate different proteins exerting sophisticated functions.^5^, ^6^, ^7^, ^8^ Common alternative splicing patterns include exon skipping, intron retention, alternative 5′ splice site, alternative 3′ splice site (A3SS) and mutually exclusive exons. These mRNA isoforms are derived from identical precursor messenger RNAs but exhibit variations in retained exon regions. Apart from the common patterns, alternative first exons or alternative promoter usage also can yield mRNA isoforms with distinct 5′ ends via the use of different transcription start sites.^9^, ^10^, ^11^, ^12^ The occurrence of isoform changes can be regulated by various factors, such as genomic epigenetic modifications.^13^, ^14^, ^15^ Since parental genomes of the zygote post‐fertilization have been reported to exhibit significant epigenetic differences,^16^, ^17^, ^18^ suggesting different mRNA isoform patterns might be generated from the paternal and maternal genomes following ZGA. To date, several studies have reported the differences in mRNA isoforms expressed before and after ZGA by using normal fertilized embryos.^19^, ^20^, ^21^, ^22^ However, whether paternal or maternal genomes generate specific patterns of mRNA isoforms during mammalian ZGA has not been reported. A systematical depiction of the mRNA isoform patterns during ZGA and between parental genomes will provide vital insights into how sperm and oocyte genomes regulate complicated mammalian development.

To address this question, we performed a comparative analysis of the transcriptomes from intracytoplasmic sperm injection (ICSI)‐derived, parthenogenetic (PG, containing only maternal genome) and androgenetic (AG, containing only paternal genome) embryos at three stages: early 2‐cell (E2C), late 2‐cell (L2C) and 4‐cell (4C) stage. Gene expression patterns during ZGA were initially assessed among normal ICSI, PG and AG mouse embryos at E2C (minor ZGA), L2C (major ZGA) and 4C (post‐ZGA) stages. A total of 18 sequenced samples were collected for time‐course RNA sequencing using the Smart‐seq3 library preparation, with a duplicate at each time point (Figure 1A). Subsequently, principal component analysis and unsupervised hierarchical clustering revealed that L2C and 4C stage embryos exhibited similar overall transcriptional profiles, distinct from those of E2C stage embryos (Figure 1B,C). This observation aligns with the understanding that major ZGA occurs during the L2C stage in mouse embryos^2^, ^3^, ^4^ and also verifies that major ZGA occurs at the same time in AG and PG embryos as that observed in normal ICSI embryos. Then, isoform differences before and after major ZGA were identified by comparing the transcriptome data of ICSI, AG and PG embryos at E2C and 4C stages using rMATS and SUPPA2.^26^, ^27^ This analysis identified 682 isoform differences, of which 118 were attributed to alternative splicing, and 564 were attributed to alternative promoter usage. To further explore the isoform patterns between parental genomes following ZGA, transcriptome data of AG and PG embryos at the 4C stage were comparatively analyzed. Notably, we identified 229 differential alternative splicing events and 337 differential alternative promoter selection events between AG and PG embryos post‐ZGA, indicating different transcriptional control between maternal and paternal genomes during ZGA. Among the splicing differences, exon skipping was the predominant splicing type observed both during ZGA (81 out of 118) and between AG and PG embryos post‐ZGA (180 out of 229) (Figure 1D; Table S1). These findings were consistent with previously reported distributions of alternative splicing event types.^28^, ^29^

**FIGURE 1:**
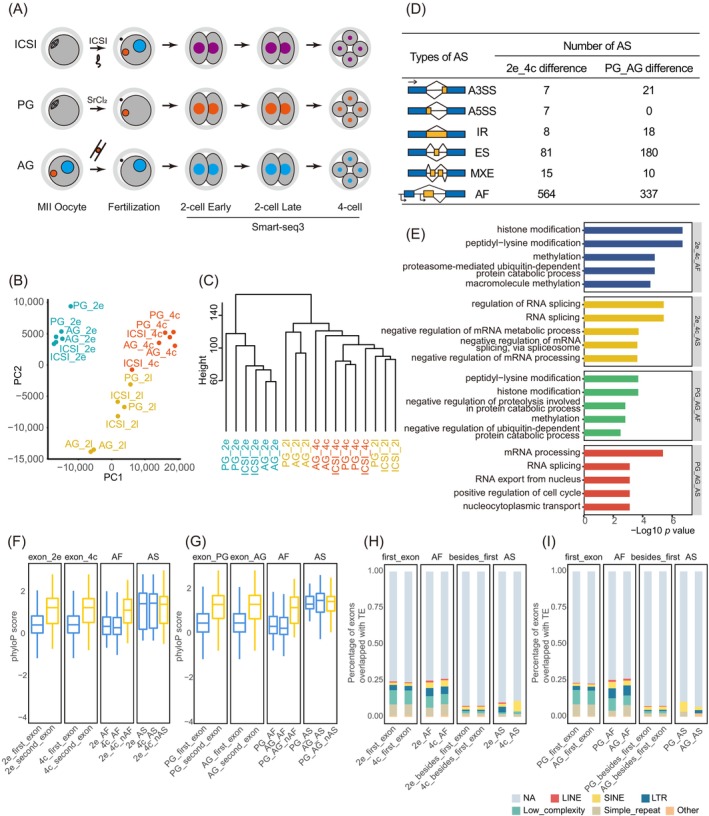
Differential alternative splicing events before and after zygotic genome activation (ZGA) and between maternal and paternalmouse embryos. (A) Schematic representation of the sample collection and sequencing protocol used in the investigation of intracytoplasmic sperm injection (ICSI), parthenogenetic (PG) and androgenetic (AG)^23^ embryonic samples. Oocytes at the MII stage were collected from B6D2F1 (C57BL/6 × DBA/2) mice, and sperm was obtained from PWK/PhJ mice. (B) Principal component analysis of the gene expression levels in embryo samples at various developmental stages. (C) Unsupervised clustering analysis of the gene expression levels in embryo samples at various developmental stages. (D) Statistical analysis of differential alternative splicing or alternative promoter usage events between different groups. The 2e, 2l, and 4c denote the early 2‐cell stage, late 2‐cell stage and 4‐cell stage, respectively. (E) Gene Ontology^24^ term enrichment analysis of genes with differential alternative splicing or alternative first exon^25^ events between before and after ZGA, as well as between PG and AG embryos. (F) Conservation of exons related to differential isoforms between pre‐ and post‐ZGA embryos, assessed using PhyloP scores. 2e_first_exon: All genes' first exons expressed in the early 2‐cell stage. 2e_second_exon: All genes' second exons expressed in the early 2‐cell stage. 2e_AF: Alternative promoter usage events preferentially used in the early 2‐cell stage. 4c_AF: Alternative promoter usage events preferentially used in the 4‐cell stage. nAF: All exons except for the first exon in one transcript. nAS: All exons except for alternative exons in one transcript. (G) Conservation of exons related to differential isoforms between PG and AG embryos, assessed using PhyloP scores. (H) Distribution of transposable elements (TEs) in exons related to differential isoforms between pre‐ and post‐ZGA embryos. (I) Distribution of TEs in exons related to differential isoforms between PG and AG embryos.

The potential functions of isoform differences were further evaluated by functional enrichment analysis between pre‐ and post‐ZGA embryos, as well as between parental genomes post‐ZGA. Notably, many alternative splicing–mediated isoform differences were associated with RNA‐binding proteins related to RNA processing and splicing. Meanwhile, alternative promoter usage–mediated isoform changes were predominantly enriched in epigenetic regulation processes, such as histone modification (Figure 1E).

Next, conservation analysis of the exons undergoing alternative splicing and alternative promoter selection during ZGA was performed using phyloP scores (Figure 1F,G). Consistent with the overall expression trends of genes in early embryos, we observed that the first exons generated by alternative promoter usage exhibited lower conservation scores than the adjacent second exons. In contrast, there was no significant conservation difference among the exons undergoing alternative splicing compared to their adjacent exons. In addition, the conservation trends of exons involved in isoform changes between parental genomes were consistent with the isoform changes observed before and after ZGA. These findings were further supported by corresponding phastCons conservation scores (Figure S1A,B).

Transposable elements are mobile DNA sequences capable of moving from one location to another within a genome. These sequences act as cis‐regulatory elements, playing a critical role in transcriptional regulation and alternative splicing. Therefore, we explored the distribution of transposable elements on exons undergoing alternative splicing or alternative promoter usage events (Figure 1H,I). Notably, a higher enrichment of transposable elements was observed on exons corresponding undergoing alternative promoter usage events, consistent with the proportion observed in all genes expressed in early embryos. However, when considering the types of transposable elements, a higher proportion of short interspersed nuclear element and long terminal repeat (LTR) elements was detected in the transposable elements enriched on exons associated with alternative promoter usage. Conversely, exons involved in alternative splicing events exhibited a lower proportion of integrated transposable elements. Moreover, a similar distribution of transposable elements, which were located on exons related to isoform differences, was observed between parental genomes.

Further analyses were subsequently conducted on their functional implications, conservation, and overlap with transposable elements. Previous studies have highlighted the involvement of transcription factors and epigenetic regulators in cell fate regulation through shifts in isoform expression.^9^, ^23^, ^30^ Thus, we investigated the isoform transitions of transcription factors and epigenetic regulators during ZGA. We identified a total of 7 transcription factors and 13 genes involved in epigenetic regulation that exhibited isoform switches before and after ZGA (Table S1).

Cold shock domain containing E1 (Csde1) is an RNA‐binding protein that contains 9 cold shock domains (CSDs) and plays complex bidirectional functions in mRNA stability and translation.^31^ We observed a significant exon skipping event on the 6th exon resulting in the loss of CSD2 of Csde1 during the ZGA (Figure 2A,B). This isoform switch was also observed when reanalyzing previously published early embryo datasets (Figure S2A). CSD2 domain of Csde1 has been found to involve in mRNA decay associated with carbon catabolite repression 4 (CCR4).^32^ CCR4‐negative on TATA‐less (NOT) complex has shown to regulate mRNA stability and participate in maternal mRNA clearance during ZGA in mice.^33^, ^34^ Given these findings, we hypothesized that a high proportion of Csde1 containing CSD2 at 2C stage might be due to the high demand on maternal mRNA degradation. A significant decline in levels of the total Csde1 and the isoform with CSD2 after ZGA also could provide the evidence for our hypothesis. Besides *Csde1*, two other genes, WD40 repeat domain protein 5 (*Wdr5*) and ENY2 transcription and export complex 2 subunit (*Eny2*), both encoding epigenetic factors possessing RNA‐binding domains,^35^, ^36^ were also found to undergo isoform switches during ZGA, leading to alterations in protein coding (Figure 2C,D and Figure S2B,C).

**FIGURE 2:**
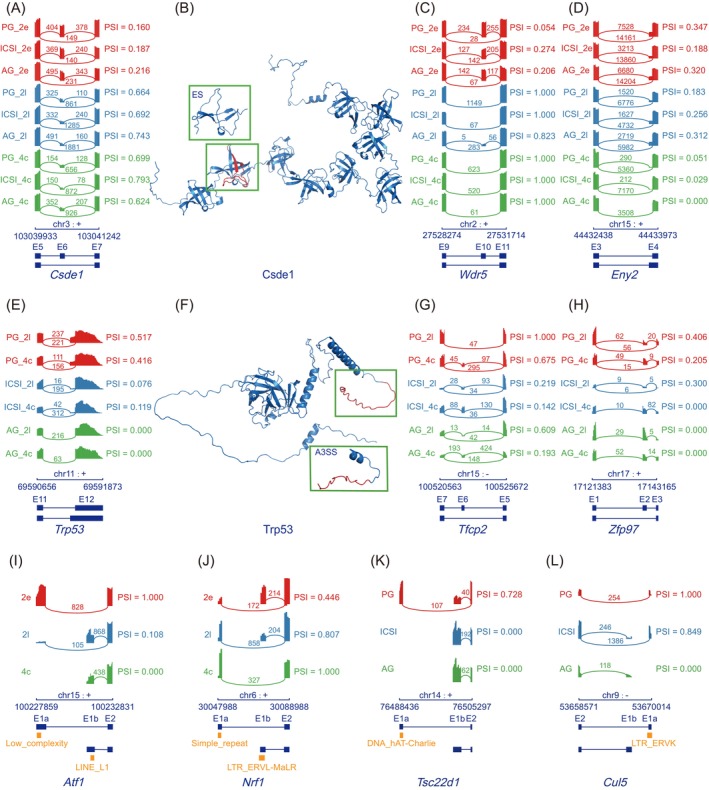
Key genes exhibiting differential alternative splicing and alternative promoter usage before and after zygotic genome activation (ZGA), as well as between parthenogenetic (PG) and androgenetic (AG) embryos. (A) Sashimi plots showing exon skipping events within the cold shock domain containing E1 (*Csde1*) gene that were differentially observed between pre‐ and post‐ZGA embryos. Percent spliced in (PSI) denotes the ratio of normalized read counts, containing the inclusion of a transcript element to the total normalized reads for that event. (B) Protein structure prediction for Csde1 isoforms before and after skipping the sixth exon, performed by ColabFold online software. ES denotes exon skipping. (C) Sashimi plots displaying exon skipping events within the *Wdr5* gene that were differentially observed between pre‐ and post‐ZGA embryos. (D) Sashimi plots of A3SS events within the ENY2 transcription and export complex 2 subunit (*Eny2*) gene that were differentially observed between pre‐ and post‐ZGA embryos. A3SS denotes alternative 3′ splice sites. (E) Sashimi plots displaying A3SS events within the tumour suppressor gene p53 (*Trp53*) gene that were differentially observed between PG and AG embryos. (F) Protein structure prediction for Trp53 isoforms with different terminal domains (CTDs), performed by ColabFold online software. A3SS denotes alternative 3′ splice sites. (G) Sashimi plots of exon skipping events within the transcription factor CP2 (*Tfcp2*) gene that were differentially observed between PG and AG embryos. (H) Sashimi plots of exon skipping events within the zinc finger protein 97 (*Zfp97*) gene that were differentially observed between PG and AG embryos. (I) Sashimi plots of alternative promoter usage events within the activating transcription factor 1 (*Atf1*) gene that were differentially observed between pre‐and post‐ZGA embryos. (J) Sashimi plots of alternative promoter usage events within the nuclear respiratory factor 1 (*Nrf1*) gene that were differentially observed between pre‐ and post‐ZGA embryos. (K) Sashimi plots of alternative promoter usage events within the TSC22 domain family member 1 (*Tsc22d1*) gene that were differentially observed between PG and AG embryos. (L) Sashimi plots of alternative promoter usage events within the cullin‐5 (*Cul5*) gene that were differentially observed between PG and AG embryos.

We also identified isoform differences in 14 transcription factors and 18 epigenetic factors between AG and PG embryos, reflecting the differences between parental transcriptional regulation during ZGA (Table S1). For example, a higher abundance of A3SS isoforms of tumour suppressor gene p53 (*Trp53*) was observed in PG embryos derived from the maternal genome, compared to transcripts derived from AG embryos at the L2C or 4C stage (Figure 2E and Figure S2D). These A3SS isoforms resulted in changes to the C‐terminal domains (CTDs) involved in site‐specific DNA binding^37^ (Figure 2F). Furthermore, transcription factor CP2 (*Tfcp2*) and zinc finger protein 97 (*Zfp97*) were also found to exhibit splicing differences between the maternal and paternal genomes, as detected in our samples and other public datasets (Figure 2G,H and Figure S2E,F).

Among the factors that displayed different alternative promoter usage between parental genomes or before and after ZGA, we identified 15 transcription factors and 29 epigenetic regulators with transposable elements in their alternative promoter regions (Table S1). For example, the cyclic AMP‐dependent transcription factor activating transcription factor 1 (*Atf1*), which plays a critical role in early embryonic development,^38^ was observed to transition from a low‐complexity repetitive sequence to a long interspersed nuclear element 1 (LINE1) element–driven transcript during the ZGA (Figure 2I and Figure S2G). However, the nuclear respiratory factor 1 (*Nrf1*) isoform initiated by an LTR element was repressed following ZGA (Figure 2J and Figure S2H). Meanwhile, when comparing isoform differences between parental genomes, we observed promoter switch events involving transposable elements in TSC22 domain family member 1 (*Tsc22d1*) and cullin‐5 (*Cul5*) genes, which act as a transcription repressor and ubiquitin ligase, respectively (Figure 2K,L and Figure S2I,J).

To further explore whether the findings from the mouse could be applicable to the humans, we analyzed differential alternative splicing and differential alternative first exon events in public human embryo datasets^24^, ^39^ by using the same analytical methodology. We discovered 454 alternative splicing and 564 alternative first exon events between pre‐ and post‐ZGA in humans (Figure S3A). Besides, we also identified 615 alternative splicing and 172 alternative first exon events between maternal‐ and paternal embryos during ZGA in humans. Comparative analysis between mice and humans showed that 441 genes covered 682 differential alternative splicing and alternative first exon events in mice, whereas 692 genes covered total 1018 differential events in humans during ZGA. Out of these genes, 62 genes were found to be overlapped in both species (Figure S3B). Additionally, compared the maternal embryos with paternal embryos during ZGA, we also found that 565 total alternative splicing events were generated from 414 genes in mice, while 787 total events were produced from 574 genes in humans. Among these genes, 17 were overlapped in both species (Figure S3C). Notably, we especially analyzed the respective isoform switch patterns of *CSDE1* and *WDR5*, since they were the most significant differential alternative splicing events. These two genes exhibited a high consistent tendency on alternative splicing patterns (Figure S3D,E), indicating the importance of our findings in understanding early embryonic development of mammals.

In summary, this study elucidated the differential mRNA isoform landscape before and after ZGA and between parental chromosomes in both mice and humans. Through the identification of changes in mRNA isoforms and subsequent functional analysis, our findings suggest the presence of regulatory processes beyond differences in mRNA abundance. This involves functional variations arising from selective splicing or promoter usage during ZGA. The identification of novel isoform changes can serve as markers for early embryonic ZGA events and provide valuable insights into the regulatory mechanisms governing early embryogenesis. To address the challenges caused by the sample acquisition, we employed Smart‐seq3 library preparation, which is specifically suitable for low‐cell input samples. Moreover, we also reanalyzed the publicly available transcriptome data from corresponding stages of early embryos as additional controls.^40^, ^41^ Although we assessed each splicing event independently for its impact on protein coding, this approach is limited by the inability to consider the interplay between splicing events within the same gene and their effects on the open reading frame of transcripts. In addition, although we have identified genes with isoform‐specific differences and predicted their potential significant roles through protein structure analysis, further functional validation is required to identify the specific functions of each isoform. Overall, our study will provide valuable insights into the impact of isoform differences on ZGA that can enhance our understanding of early embryogenesis in mammals.

## AUTHOR CONTRIBUTIONS

**Fan Li:** Methodology; data curation; formal analysis; methodology; software; validation; visualization; writing—original draft. **Najmeh Karimi:** Conceptualization; formal analysis; writing—original draft. **Siqi Wang:** Conceptualization; writing—original draft; writing—review & editing. **Tianshi Pan:** Data curation; formal analysis; visualization; writing—original draft. **Jingxi Dong:** Data curation; methodology. **Xin Wang:** Data curation; methodology. **Sinan Ma:** Resources; formal analysis. **Qingtong Shan:** Resources; formal analysis. **Chao Liu:** Resources; formal analysis. **Ying Zhang:** Writing—review and editing; supervision. **Wei Li:** Conceptualization; funding acquisition; project administration; writing—review and editing. **Guihai Feng:** Conceptualization; funding acquisition; investigation; project administration; supervision; writing—original draft; writing—review and editing. All authors have read and approved the final manuscript.

## FUNDING INFORMATION

This work was supported by the National Key Research and Development Program of China (2019YFA0110901, 2022YFA1104101, 2022YFA1104302, 2022YFA0806302, 2021YFA0719303), Initiative Scientific Research Program, Institute of Zoology, Chinese Academy of Sciences (2023IOZ0302), Informatization Plan of Chinese Academy of Sciences (CAS‐WX2021SF‐0101).

## CONFLICT OF INTEREST STATEMENT

The authors declare no conflicts of interests.

## Supporting information


**Data S1.** Supporting Information.

**Table S1.** Detailed information on the differential alternative splicing and alternative promoter usage events before and after ZGA, as well as between PG and AG embryos.
