# Supplementary material for: mRNA isoform switches during mouse zygotic genome activation
Source: Cell Prolif. 2024 May 19;57(7):e13655. doi: 10.1111/cpr.13655 (PMC11216927; doi:10.1111/cpr.13655)
Supplement: Supplementary file 1 — Data S1. Supporting Information. [file CPR-57-e13655-s001.docx]

**Supplementary Files**

**Supplementary Figure 1**

**
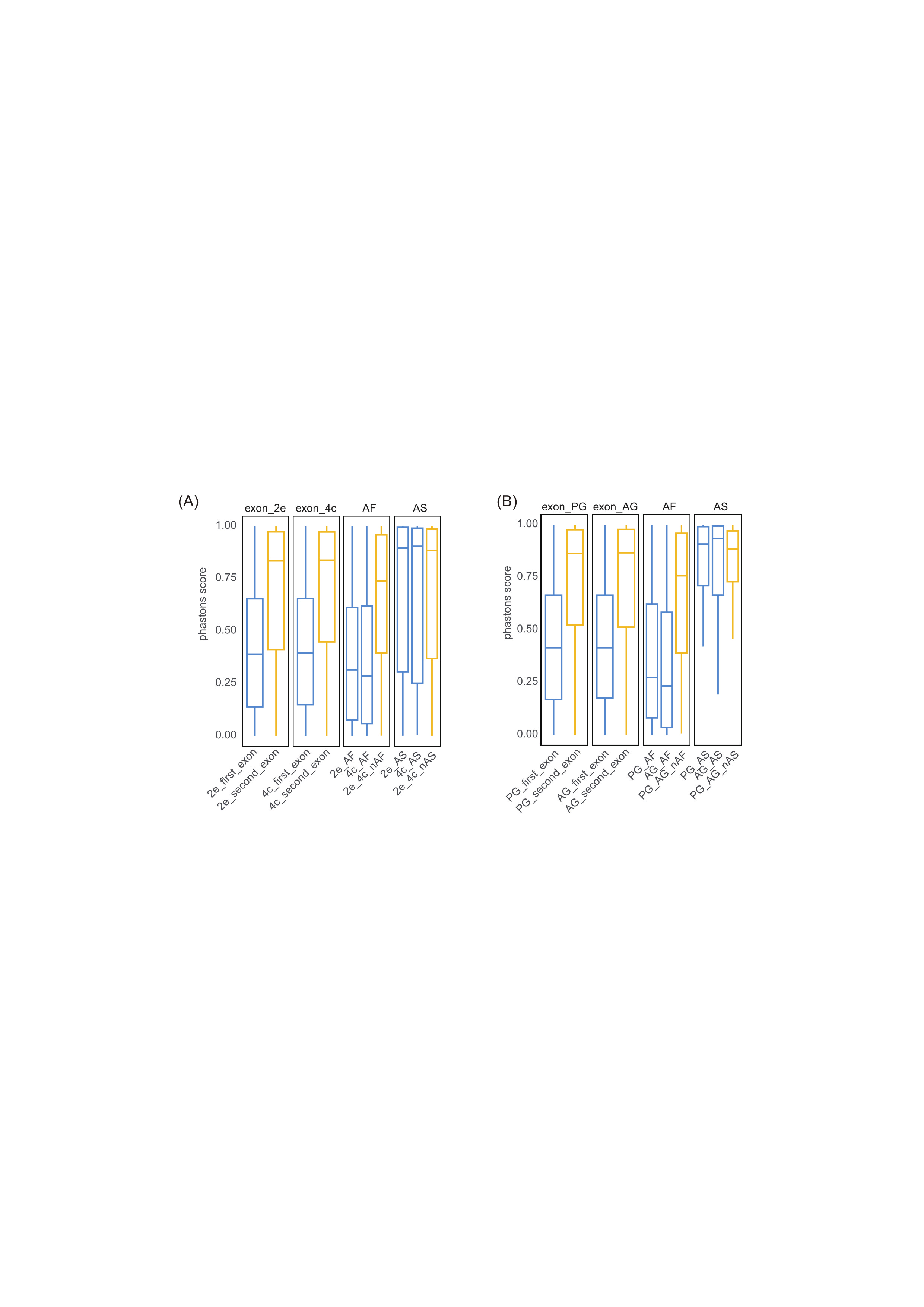
**

**Figure S1** Exon conservation analysis using PhastCons scores. (A) Conservation of exons related to differential isoforms between pre- and post-ZGA embryos, assessed using PhastCons scores. (B) Conservation of exons related to differential isoforms between PG and AG embryos, assessed using PhastCons scores.

**Supplementary Figure 2**


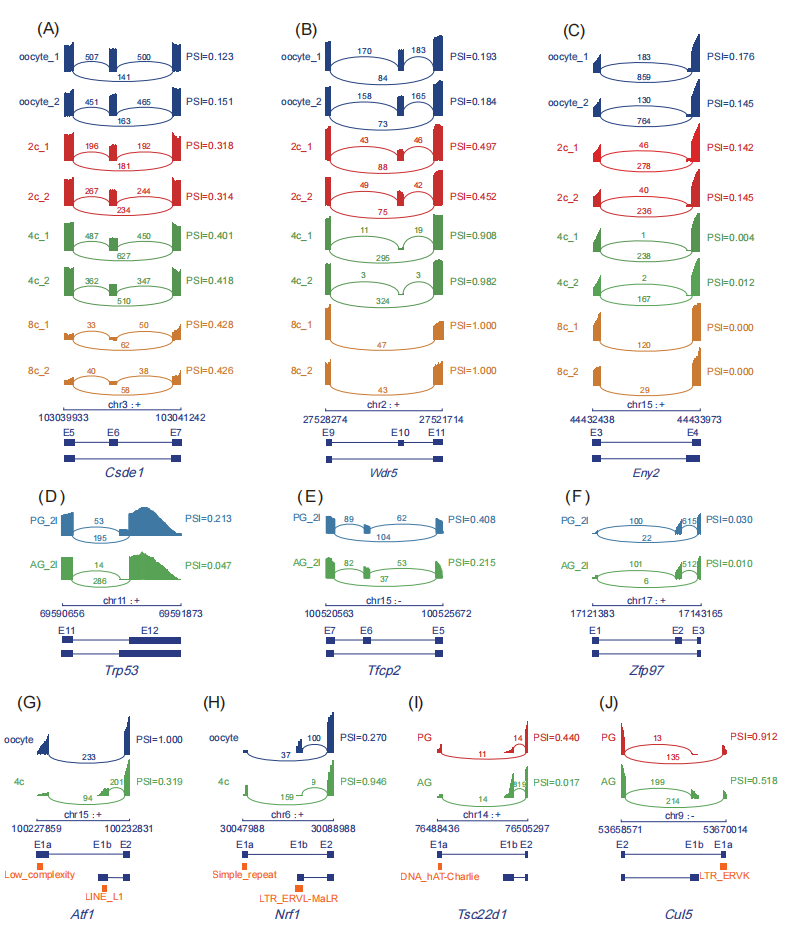


**Figure S2** Sashimi plots of alternative splicing or alternative promoter usage events identified before and after ZGA, as well as between PG and AG embryos using publicly available datasets. (A) Sashimi plots of exon skipping events within the *Csde1* gene that were differentially observed between pre- and post-ZGA embryos. (B) Sashimi plots of exon skipping events within the *Wdr5* gene that were differentially observed between pre- and post-ZGA embryos. (C) Sashimi plots of alternative 3′ splice site events within the *Eny2* gene that were differentially observed between pre- and post-ZGA embryos. (D) Sashimi plots of alternative 3′ splice site events within the *Trp53* gene that were differentially observed between PG and AG embryos. (E) Sashimi plots of exon skipping events within the *Tfcp2* gene that were differentially observed between PG and AG embryos. (F) Sashimi plots of exon skipping events within the *Zfp97* gene that were differentially observed between PG and AG embryos. (G) Sashimi plots of alternative promoter usage events within the *Atf1* gene that were differentially observed between pre- and post-ZGA embryos. (H) Sashimi plots of alternative promoter usage events within the *Nrf1* gene that were differentially observed between pre- and post-ZGA embryos. (I) Sashimi plots of alternative promoter usage events within the *Tsc22d1* gene that were differentially observed between PG and AG embryos. (J) Sashimi plots of alternative promoter usage events within the Cul5 gene that were differentially observed between PG and AG embryos.

**Supplementary Figure 3**


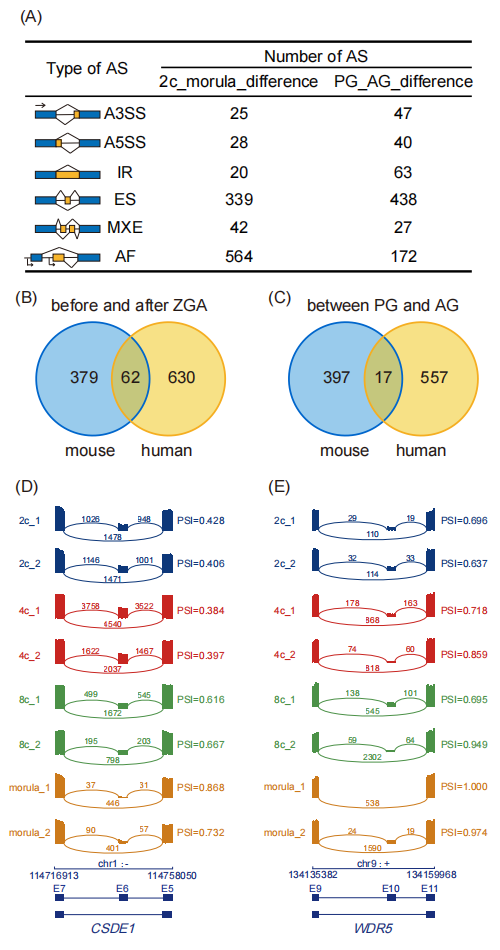


**Figure S3** Differential alternative splicing events before and after ZGA, and between maternal- and paternal-derived embryos using human publicly available datasets. (A) Statistical analysis of differential alternative splicing or alternative promoter usage events between different groups. The 2c denote the 2-cell stage. (B) Number of genes producing differential alternative splicing events or alternative promoter usage events before and after ZGA in mice and humans. 62 genes were overlapped. (C) Number of genes producing differential alternative splicing events or alternative promoter usage events between maternal- and paternal-derived embryos in mice and humans. 17 genes were overlapped. (D) Sashimi plots of exon skipping events within the *CSDE1* gene that were differentially observed between pre- and post-ZGA in human embryos. (E) Sashimi plots of exon skipping events within the *WDR5* gene that were differentially observed between pre- and post-ZGA in human embryos. These two differential splicing events have a consistent tendency with what happened in mice.

**Table S1. Detailed information on the differential alternative splicing and alternative promoter usage events before and after ZGA, as well as between PG and AG embryos. （TableS1.xlsx excel file）**

**Supplementary Methods and Materials**

***Animals***

C57BL/6 and DBA/2 mice were purchased from Beijing Vital River Laboratory Animal Technology (China). PWK/PhJ mice were purchased from Jackson Laboratory. Female B6D2F1 mice (C57BL/6 × DBA/2) were used for oocyte collection. Mice were kept on a 12 h light cycle of 8 am to 8 pm, with *ad libitum* access to food and water. All animal studies were performed in accordance with the Guidelines for the Use of Animals in Research issued by the Institute of Zoology, Chinese Academy of Sciences.

***Oocyte collection***

Eight-week-old female mice were subjected to super-ovulation via intraperitoneal injection of 5 IU of pregnant mare serum gonadotropin followed by 5 IU of human chorionic gonadotropin (hCG) 48 h later. Subsequently, the mice were euthanized by cervical dislocation, and oocyte corona cumulus complexes (OCCOs) were collected from the ampullae of oviducts 13–15 h after hCG injection [1]. These OCCOs were then transferred to human tubal fluid medium supplemented with 10 mg/mL BSA. Cumulus cells were dissociated by treatment with the OCCOs and 300 µg/mL hyaluronidase in M2 media, combined with gentle pipetting for 60 s. The resulting oocytes were washed with HEPES-CZB and cultured in M16 medium. Before micromanipulation, the oocytes were cultured in CZB medium supplemented with 3 mg/mL BSA at 37°C and 5% CO_2_.

***Embryo derivation and culture***

Sexually mature male PWK/PhJ mice were euthanized to obtain spermatozoa. Three groups of embryos were prepared, including normal, PG and AG embryos. Briefly, normal embryos were prepared using ICSI. MII oocytes were placed into a drop of HCZB medium; then, a single sperm head was injected into each MII oocyte using a piezo impact-driven micromanipulator. The resulting embryos were then cultured in KSOM-AA medium at 37°C in a 5% CO_2_ environment. To generate PG embryos, oocytes were incubated in activating media containing 10 mM SrCl_2_, with 3 h exposure at 37.5°C in a humidified atmosphere with 5% CO_2_. Following SrCl_2_ treatment, the oocytes were transferred to regular CZB (Chatot, Ziomek, and Bavister) medium. Six hours after the onset of activation treatment with SrCl_2_, the oocytes were examined under a microscope for evidence of activation, indicated by the presence of one pronucleus (1PN) or two well-developed pronuclei (2PN). Finally, AG embryos were produced by transferring zygotes to M2 media containing 5 μg/mL cytochalasin B following 7 hours of PWK/PhJ sperm injection. Then, female pronuclei were removed using a piezo impact-driven micromanipulator. Female pronuclei, identified based on size and distance from the polar body, were individually extracted from the zygotes at stages PN3-4 by piercing the zona pellucida using a Piezo drive (Prime Tech) and aspirating with a micromanipulator [2]. Zygotes containing a male pronucleus were cultured in KSOM with amino acids at 37°C under 5% CO_2_. All normal, PG, and AG embryos at the E2C, L2C, and 4C stages were collected at 24, 36, and 44 h after insemination, respectively, with each group consisting of 10 embryos. This procedure was performed in triplicate.

***Smart-Seq3 library preparation and sequencing***

As previously described [3], embryo samples were collected and placed in lysis buffer containing RNase, RNase inhibitor, Triton X-100, oligo-dT, dNTP, and PEG8000. Following lysis, single-strand cDNA synthesis was performed using a reverse transcription reaction system comprising reverse transcriptase, RNase inhibitor, Betaine, single-strand buffer, DTT, magnesium chloride, TSO, GTP, NaCl_2_, and nuclease-free water. The reaction protocol included incubation at 42°C for 90 min, followed by 10 cycles at 50°C for 2 minutes and 42°C for 2 minutes, a final extension at 85°C for 5 minutes, and holding at 4°C. The resulting reverse transcription products served as templates for PCR amplification.

The PCR reaction system consisted of polymerase, PCR buffer, dNTP, primers, and nuclease-free water. PCR amplification was performed with an initial denaturation step at 98°C for 3 minutes, followed by 20 cycles at 98°C for 20 seconds, 65°C for 30 seconds, and 72°C for 4 minutes. A final extension step was then conducted at 72°C for 5 minutes, followed by holding the reaction at 4°C. Subsequently, the PCR products were purified using AMPure XP magnetic beads, and the final purified product was dissolved in EB buffer. The quality of the amplified products was assessed using Fragment Analyzer 1.0.2.9, focusing on fragment size distribution > 1 kb.

For library construction, the KAPA Hyper Prep Kits (KK8504) were employed. This process involved cDNA fragmentation via incubation at 55°C for 10 minutes. PCR amplification was then conducted with thermal cycling steps as follows: 105°C for 3 minutes, denaturation at 98°C for 30 seconds, and 5–15 cycles of 98°C for 15 seconds, 60°C for 30 seconds, and 72°C for 3 minutes. Following PCR amplification, AMPure XP magnetic beads were utilized for size selection and purification of the fragments. Library size was assessed using Fragment Analyzer 1.0.2.9, with the fragment size distribution predominantly ranging from 300 to 700 bp. Quantification of the libraries was performed using Qubit. Finally, the libraries were sequenced using the Illumina Novaseq6000 platform with a PE150 (paired-end 150) configuration.

***Sequencing data analysis***

Low-quality reads and adapter sequences were removed from the sequencing data using TrimGalore (Krueger et al., 2021, GitHub repository, https://github.com/FelixKrueger/TrimGalore). The aligned sequencing data was then mapped to the mm10 reference genome using STAR [4]. Gene and transcript abundance were quantified using StringTie [5]. To normalize gene expression levels, Fragments Per Kilobase of transcript per Million mapped fragments (FPKM) was employed. Genes with FPKM values > 1 in at least one sample were selected for further analysis.

Principal component analysis was performed on the samples using FactoMineR [6], with the results being visualized using ggrepel [7] and factoextra. Hierarchical clustering of all samples was conducted using the hclust function [8] with the complete linkage method for calculating distances between clusters. For Gene Ontology (GO) enrichment analysis, the clusterProfiler package [9] in R was utilized. The statistical significance threshold for GO enrichment analyses was set at P-value < 0.05.

***Differential alternative splicing event analysis***

To identify differential alternative splicing events between the E2C and 4C samples, duplicate sample BAM files were merged using samtools [10]. The samples ICSI_E2C, PG_E2C, and AG_E2C were treated as three replicates, while ICSI_4C, PG_4C, and AG_4C served as three replicates. rMATS [11] software was employed to analyze five types of alternative splicing events: A3SS, A5SS, RI, SE, and MXE. Differential splicing events between the E2C and 4C samples were identified based on the criteria of ΔPSI > 0.1, P-value < 0.05, and sum of read counts > 10. Next, the BAM files for ICSI_E2C, PG_E2C, and AG_E2C were merged using samtools. Similarly, the BAM files for ICSI_L2C, PG_L2C, and AG_L2C were merged, and the BAM files for ICSI_4C, PG_4C, and AG_4C were merged. Next, SUPPA2 [12] software was employed to analyze AF events. AF events that met the following criteria were considered as differential AF events between the E2C and 4C samples: ΔPSI > 0.1 and P-value < 0.05.

To analyze differential splicing events between PG and AG samples during the L2C and 4C stages, rMATS software was utilized. This analysis focused on the five previously specified types of differential splicing events. The criteria used for identifying differential splicing events were as follows: ΔPSI > 0.1, P-value < 0.05, and a sum of read counts > 10. Additionally, it was necessary to ensure the PSI_ICSI value fell between PSI_PG and PSI_AG, with consistency in the differential splicing events between the L2C and 4C stages. For the analysis of differential AF events between PG and AG samples during the 4C stage, SUPPA2 was employed. Next, stringtie was used to merge the transcript file of the mm10 reference genome with the newly acquired transcript file obtained by Qiao et al [13]. The criteria used to identify differential AF events between PG and AG samples included ΔPSI > 0.1, P-value < 0.05, and a PSI_ICSI value between PSI_PG and PSI_AG.

***Other publicly available resources and tools***

The phastCons60wayPlacental and phyloP60wayPlacental files obtained from the UCSC database were employed to calculate the evolutionary conservation scores for the exons involved in alternative splicing events and their adjacent regions [14, 15]. The transposon elements annotated by RepeatMasker were utilized to assess the distribution of transposon elements in the exons involved in alternative splicing and their adjacent exons (A.F.A. Smit, R. Hubley & P. Green, http://repeatmasker.org). In instances where an exon overlapped with multiple transposon elements, only the first overlap was considered for counting. Transcription factor annotation was downloaded from the AnimalTFDB3 database [16], while the epigenetic factor annotation was obtained from the Epifactors database [17]. Protein structures were predicted using the default parameters on the ColabFold online website [18]. Public datasets GSE98150 from the Gene Expression Omnibus database and CRA005750 from the Genome Sequence Archive were downloaded to analyze the differential alternative splicing events before and after ZGA or between AG and PG embryos, respectively [19, 20].

Public datasets GSE36552 [21] and GSE133854 [22] from the Gene Expression Omnibus database were downloaded to analyze the differential alternative splicing events before and after ZGA or between AG and PG embryos in human, respectively. In the analysis of human differential alternative splicing events, we adopted stricter criteria. To identify differential alternative splicing events before and after ZGA, we combined the 2-cell stage and morula-stage single blastomere data separately using samtools. Differential splicing events between the 2C samples and morula samples were identified based on the criteria of ΔPSI > 0.4, P-value < 0.05, sum of read counts > 15, and read counts that supports the event > 5. Differential AF events between the 2C samples and morula samples were identified based on the criteria of ΔPSI > 0.4 and P-value < 0.05. To identify differential alternative splicing events between paternal and maternal-derived embryos, we combined the morula-stage single blastomere data for paternal and maternal-derived embryos, separately. Filtering was then performed with the same thresholds. To observe whether there is a consistent trend in differentially splicing events between humans and mice, we used ggvenn to perform overlap analyses of genes with differential splicing events in humans and mice before and after ZGA, and between paternal and maternal-derived embryos during ZGA.

**References**

1. Lawitts, J.A. and J.D. Biggers, *Culture of preimplantation embryos.* Methods Enzymol, 1993. **225**: p. 153-64.

2. Gu, T.P., et al., *The role of Tet3 DNA dioxygenase in epigenetic reprogramming by oocytes.* Nature, 2011. **477**(7366): p. 606-10.

3. Hagemann-Jensen, M., et al., *Single-cell RNA counting at allele and isoform resolution using Smart-seq3.* Nat Biotechnol, 2020. **38**(6): p. 708-714.

4. Dobin, A., et al., *STAR: ultrafast universal RNA-seq aligner.* Bioinformatics, 2013. **29**(1): p. 15-21.

5. Pertea, M., et al., *StringTie enables improved reconstruction of a transcriptome from RNA-seq reads.* Nat Biotechnol, 2015. **33**(3): p. 290-5.

6. Lê, S., J. Josse, and F. Husson, *FactoMineR: An R Package for Multivariate Analysis.* Journal of Statistical Software, 2008. **25**(1): p. 1 - 18.

7. Wilkinson, L., *ggplot2: Elegant Graphics for Data Analysis by WICKHAM, H.* Biometrics, 2011. **67**(2): p. 678-679.

8. Langfelder, P. and S. Horvath, *Fast R Functions for Robust Correlations and Hierarchical Clustering.* J Stat Softw, 2012. **46**(11).

9. Wu, T., et al., *clusterProfiler 4.0: A universal enrichment tool for interpreting omics data.* Innovation (Camb), 2021. **2**(3): p. 100141.

10. Danecek, P., et al., *Twelve years of SAMtools and BCFtools.* Gigascience, 2021. **10**(2).

11. Shen, S., et al., *rMATS: robust and flexible detection of differential alternative splicing from replicate RNA-Seq data.* Proc Natl Acad Sci U S A, 2014. **111**(51): p. E5593-601.

12. Trincado, J.L., et al., *SUPPA2: fast, accurate, and uncertainty-aware differential splicing analysis across multiple conditions.* Genome Biol, 2018. **19**(1): p. 40.

13. Qiao, Y., et al., *High-resolution annotation of the mouse preimplantation embryo transcriptome using long-read sequencing.* Nat Commun, 2020. **11**(1): p. 2653.

14. Siepel, A., et al., *Evolutionarily conserved elements in vertebrate, insect, worm, and yeast genomes.* Genome Res, 2005. **15**(8): p. 1034-50.

15. Pollard, K.S., et al., *Detection of nonneutral substitution rates on mammalian phylogenies.* Genome Res, 2010. **20**(1): p. 110-21.

16. Hu, H., et al., *AnimalTFDB 3.0: a comprehensive resource for annotation and prediction of animal transcription factors.* Nucleic Acids Res, 2019. **47**(D1): p. D33-D38.

17. Marakulina, D., et al., *EpiFactors 2022: expansion and enhancement of a curated database of human epigenetic factors and complexes.* Nucleic Acids Res, 2023. **51**(D1): p. D564-D570.

18. Mirdita, M., et al., *ColabFold: making protein folding accessible to all.* Nat Methods, 2022. **19**(6): p. 679-682.

19. Yuan, S., et al., *Human zygotic genome activation is initiated from paternal genome.* Cell Discov, 2023. **9**(1): p. 13.

20. Wang, C., et al., *Reprogramming of H3K9me3-dependent heterochromatin during mammalian embryo development.* Nat Cell Biol, 2018. **20**(5): p. 620-631.

21. Yan, L., et al., *Single-cell RNA-Seq profiling of human preimplantation embryos and embryonic stem cells.* Nat Struct Mol Biol, 2013. **20**(9): p. 1131-9.

22. Leng, L., et al., *Single-Cell Transcriptome Analysis of Uniparental Embryos Reveals Parent-of-Origin Effects on Human Preimplantation Development.* Cell Stem Cell, 2019. **25**(5): p. 697-712.e6.
